# Tests for finding complex patterns of differential expression in cancers: towards individualized medicine

**DOI:** 10.1186/1471-2105-5-110

**Published:** 2004-08-12

**Authors:** James Lyons-Weiler, Satish Patel, Michael J Becich, Tony E Godfrey

**Affiliations:** 1 Department of Pathology, Center for Biomedical Informatics, and Interdisciplinary Biomedical Graduate Program, University of Pittsburgh, PA 15232 USA; 2 Clinical Genomics Facility, Center for Pathology Informatics, Benedum Center for Oncology Informatics, University of Pittsburgh Cancer Institute, Pittsburgh, PA 15232 USA; 3 Departments of Surgery and Human Genetics, University of Pittsburgh Medical School, Pittsburgh, PA 15232 USA; 4 Mount Sinai School of Medicine, One Gustave Levy Place, Box 1668, East Building, Room 1070C, New York, NY 10029 USA

## Abstract

**Background:**

Microarray studies in cancer compare expression levels between two or more sample groups on thousands of genes. Data analysis follows a population-level approach (e.g., comparison of sample means) to identify differentially expressed genes. This leads to the discovery of 'population-level' markers, i.e., genes with the expression patterns A > B and B > A. We introduce the PPST test that identifies genes where a significantly large subset of cases exhibit expression values beyond upper and lower thresholds observed in the control samples.

**Results:**

Interestingly, the test identifies A > B and B < A pattern genes that are missed by population-level approaches, such as the t-test, and many genes that exhibit both significant overexpression and significant underexpression in statistically significantly large subsets of cancer patients (ABA pattern genes). These patterns tend to show distributions that are unique to individual genes, and are aptly visualized in a 'gene expression pattern grid'. The low degree of among-gene correlations in these genes suggests unique underlying genomic pathologies and high degree of unique tumor-specific differential expression. We compare the PPST and the ABA test to the parametric and non-parametric t-test by analyzing two independently published data sets from studies of progression in astrocytoma.

**Conclusions:**

The PPST test resulted findings similar to the nonparametric t-test with higher self-consistency. These tests and the gene expression pattern grid may be useful for the identification of therapeutic targets and diagnostic or prognostic markers that are present only in subsets of cancer patients, and provide a more complete portrait of differential expression in cancer.

## Background

Studies of differential expression of individual genes often find genes that are up-regulated in some tumors, and down-regulated in others. Microarray studies typically seek to identify differentially expressed genes using use fold-change [1], t-tests [2], and models [3-6]. Studies of global gene expression patterns in cancer have focused largely on the identification of novel cancer subtypes via classification [7-13] or the identification of differentially expressed genes [14-18]. Such studies typically use fold-change [1], t-tests [2], and models [3-6]. The methods of analysis for identifying differentially expressed genes in data from microarray experiments vary widely [20-45], but all are focused on the question of whether genes are over-expressed or under-expressed in samples in group A (e.g., tumor, or treatment, or metastastic, or responder) compared to samples in group B (e.g., normal, or control, or quiescient, or nonresponder). These patterns can efficiently be referred to as AB (overexpressed in A) and BA (underexpressed in A) patterns. Typically, researchers use study designs that favor biological replication to maximize the ability to detect reproducibly genes that are differentially expressed in a patient population, at a sacrifice of the ability to detect individual-specific patterns of differential expression with technical replication. Most cancers are diseases with heterogeneous etiologies; moreover, the development of every primary tumor in different individuals is a unique biological event. Thus, the expression levels of genes in the individual patient are also important; some important gene dysregulation may be highly specific to each individual. Statistical methods that average gene expression may hide important expressotypes (expression phenotypes). Current tests that compare mean group expression intensities are not likely to find genes that are in fact significantly dysregulated in only a proportion of the individuals in the case population, unless the magnitude of differential expression is very high in the subset of individuals. Unsupervised clustering can be used to attempt to identify unknown partitions, or subgroups within patients, but clustering is not a well-defined method for finding differentially expressed genes, and, upon discovery of novel groups, researchers are restricted to comparing group means, and cannot identify genes that may be dysregulated in subsets of patients where the combined patterns of dysregulation patterns do not suggest coherent subgroups.

## Results

**Figure 1:**
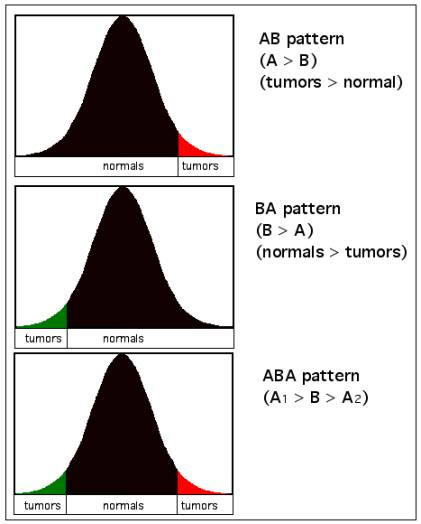
Conceptual representation of AB, BA, and ABA patterns of differential expression. The colored tails represent the placement of expression values of a given gene in tumors when compared to the distribution of expression values in normal samples. Standard AB and BA patterns are represented by red and black, respectively. Cases in which a surprising number of samples are distributed in *both *tails for a given gene are represented here as green (BA) and red (AB), respectively, and are painted similarly in Fig. 2 for specific samples.

A remarkable pattern emerges when the PPST test is applied to published cancer data sets, including breast cancer [7], ovarian cancer [16], colon cancer (epithelial-rich normals only [17,47]), lymphoma [18], and lung cancer [19] at the 99th percentile. We find an abundance of AB and BA pattern genes, with roughly the same number of genes called significant under the parametric t-test. We also find large numbers of genes with significant ABA test scores, and some with 'BAB' pattern genes (Table 1). There is a marked tendency in most data sets for more ABA (cancer-normal-cancer) type genes than BAB pattern genes. These patterns are also reflected in 'expression pattern grids' of gene with significant *s3*(ABA) statistics (Fig. 1). These patterns are reproducible at more stringent levels of α (Table 1).

The capability of the PPST test to identify genes that are in fact differentially expressed in only a subset of patients is made evident by a comparison of genes that are found to be significant under the PPST test, but missed by, for example, the t-test (even without Bonferroni-type adjustments). These are listed in Table 2, for the lymphoma data18, and notably include B-cell growth factor 1 (IL4; ABA pattern). Furthermore, 'classic' oncogenes such as cyclin D1 are found by the PPST test in the lung cancer data set [19] are not reported to be significant by the t-test. Cyclin D1 ranks 1009th among significant genes in the colon cancer data set under the t-test but ranks 90th under the PPST test (AB/BA pattern results only).

## Discussion

Our initial results are compelling in that they suggest that we can expect biomarkers of high clinical significance for subsets of patients to be important for distinct subsets of patients. This also suggests that clinical validation of the utility of biomarkers should examine panels of expression biomarkers, not individual biomarkers. Disruption of genomic function via these patterns cannot be studied in the population level biomarker framework for the simple reason that methods that compare, say, group means, will find no difference between the sample groups if the number of case samples found in the two tails are even approximately equal. This is a sensible approach even from within the framework of population-based hypothesis testing, because the PPST test can be expected to be more robust to one or two outliers that might mislead simple parametric tests. Note that a number of genes are 'nearly significant' under the t-test but are strongly significant under the PPST test for the AB/BA patterns (e.g., Table 2).

Our re-analysis of two independently generated data sets on astrocytoma progression demonstrates the utility of extending analysis to include a search for genes that are differentially expressed in a subset of patients. Of the tests examined, the parametric t-test showed the least internal consistency, while the PPST exhibited the highest internal consistency in identifying progression markers. Comparison to the non-parametric t-tests demonstrates that PPST is most similar to the nonparameteric t-test, but is more self-consistent. While the ABA test showed the least internal consistency across populations, it also exhibited low overlap with any other test, so the genes reported are unique and tend not to be found by others tests, matching expectations.

Our results are consistent with Knudsen's 'two-hit' hypothesis on the genomic etiologies of cancer [49] with some insight into the diversity of genomic pathologies (functional 'hits') that may be relevant in patient populations. Studies of differential gene expression – and its role in the etiology of cancer and its responses to treatment – should seek these types of genes in addition to population-wide biomarkers, because they represent a subset of the genes that are expressed differentially in a significant subset of cancer patients. We recommend a major shift in perspective on the study on gene expression dysregulation away from the study of 'tumor populations' – which do not exist – toward the study of genomic pathologies in individual patients. For example, tumor subtypes are typically characterized by morphological characters, and these classifications may conflict with important expressotype subtypes that do not follow classical morphological tumor classes. Imposition of these subtypes on a study design may interfere with identifying expressotypes that provide high diagnostic, prognostic and theranostic value to the individual – and sets of individuals with similar expressotypes. This view is also consistent with the Hanahan-Weinberg model of oncogenesis [50], which envisions multiple possible mechanistic strategies to the acquisition of characteristics and capabilities of cancers including self-sufficiency in growth signals, insensitivity to anti-growth signals, tissue invasion & metastasis, limitless replicative potential, sustained angiogenesis and evasion of apoptosis. We also expect that individual cancers in different patients will be found to have evolved unique sets of solutions to each of these problems. Current prevailing methods for finding differentially expressed genes such as fold-change and t-tests do not allow for such complexities.

Our comparison of the methods (Table 3) highlights the uniqueness of the ABA test. It is an extension of the PPST test; it specifically focuses on genes that are differentially expressed in subsets of patients. This ability is extremely important in search of genes with expression patterns that correlate with drug response. The ABA and the two-tailed t-test are not comparable because the ABA test allows us to find genes that the t-test specifically cannot (genes that are simultaneously overexpressed in some patients while underexpressed in others). Such test will have high variance (leading to a low t-test score) and low mean difference, and will thus not be significant. The PPST and the ABA tests extend our abilities beyond the t-test. Other improvements or even superior alternatives to these tests may be possible. The performance of these tests and all tests described to date for the AB type patterns and now for ABA patterns should be compared using extensive numerical simulations and cross-validation. Developments are needed to determine how best to select a threshold to allow deliberate control of the false positive and false negative error rates.

## Conclusions

The two major conclusions these results suggest are (1) that the most commonly applied tests for identifying differentially expressed genes will miss important genes that are dysregulated in only a fraction of patients, and (2) that important aspects of differential expression may be, to a degree, highly individualistic in most cancers. Some potentially important genes with this form of unusual differential expression (ABA; Table 2) would be missed by methods that compare group means, because the means of the two sample groups would be approximately identical, and the variance in tumors would be high, leading to a large error term. The high internal consistency of PPST compared to the non-parametric t-test and our observation that the PPST test exhibited high consistency with the nonparametric t-test suggests that the PPST test may be of interest to researchers interested in identifying both population-level biomarkers and biomarkers important to a subset of patients.

An online implementation of this test, it source code (Java), and that for many other methods of analysis, are accessible online in the Cancer Gene Expression Data Analysis tool . It is hoped that the development and application of more approaches like this will lead to a more complete representation of differential expression, leading to more meaningful and specific hypotheses of dysregulation, and thus a better comprehension of how diverse genomic pathologies contribute to the etiologies of cancers, and thereby facilitate the identification of targets that may lead to individual-specific therapies.

## Methods

We have developed a test we call the Permutation Percentile Separability Test (PPST), which attempts to refute a null hypothesis that is slightly different from A = B, but which is capable of detecting AB, BA, ABA and BAB patterns. Under this test, we are interested in the question "are there are statistically significant number of samples in group A (e.g., tumor) that exhibit expression intensities beyond a particular percentile of the observed expression intensities in group B (e.g., normal)?" and vice versa. By 'statistically significant' we mean that the number of samples that exhibit apparent overexpression (or underexpression) exceeds that expected under the null distribution.

To test these hypotheses, we count the number of samples in both groups that are found beyond the n^*th *^percentile of the samples in the opposite group. This provides two scores, *s*_1_, and *s*_2_, for each gene (PPST scores). *s*_1 _is the number of samples in group A that are beyond the upper percentile (say, 95^th^) of group B plus the number of samples in group B that are below the lower 95^th ^percentile of group A. This measure will tend to be large when all samples in both groups are significantly distinct from the alternate group in the same way (comparisons consistent with A > B). It can also be significant when a surprising number of samples in only one group varies from the expression levels in the alternate group. *s*_2 _is the number of samples with correspondingly opposite pattern (comparisons consistent with B > A). Sample class label permutations are used to generate an arbitrarily large number of permuted data sets. These scores *s*_1 _and *s*_2 _are calculated in each permuted data set to produce unique null distributions for each gene. For the sake of convenience of interpretation, we use -*s*_2 _when reporting *s*_2 _to denote underexpression. Genes with values of *s*_1 _beyond the specified acceptable Type 1 error risk (e.g., α = 5%) are determined to be significantly overexpressed in sample group A relative to B. *Individuals *in sample group A with expression intensity *values *over the 95^th ^percentile of sample group B for a given gene may be considered overexpressed. Similarly, genes with values of *s*_2 _beyond the specified Type 1 risk for *s*_2 _are deemed underexpressed in sample group B relative to A. Varying the percentile threshold allows direct control over the false discovery rate.

### Test for ABA patterns (ABA Test)

Genes that exhibit *both *significant *s*_1 _and *s*_2 _scores in this comparison may be considered 'ABA pattern genes' (Fig. 1); however, for stronger inference, permutation tests are also used to calculate *s*_3_, to determine, *for a given gene*, the number of samples from one group (A) that can expected to be distributed both in the upper and lower *n*^*th *^percentile tails of the intensity distribution *of that gene *in the other group (B); i.e., in the ABA (*s*_3_) or BAB (*s*_4_) pattern. These scores are not redundant to but rather allow for exploration of distribution-wise (upper and lower) false discovery rates. The application of the PPST test to find ABA patterns is called the 'ABA' test. Under the ABA test, differential expression of a gene may be deemed to be significant in both directions at once, i.e., simultaneously significantly over-expressed and under-expressed in a surprising number of patients in the case population. Both the PPST test and the ABA test will perform optimally when the variation in expression intensities in the normal sample population is well characterized.

A collection of published microarray data sets we have placed 'on-tap' in the caGEDA (Gene Expression Data Analysis) web application [51] were subjected to the PPST test and the ABA test. To avoid idiosyncracies that can result from the study of extreme values, we ran the tests at a fairly relaxed Type 1 error risk (α = 0.05 in both tails, or α = 0.10 overall). To compare the self-consistency of the parametric t-test, the nonparametric t-test, the PPST test and the ABA test, we re-analyzed two published data sets from independent astrocytoma progression studies [52,53]. Details of these studies are available in the original papers. In brief, Khatua et al. [52] studied global gene expression profiles from 6 early stage and 7 late-stage astrocytoma patients, while van den boom et al. [53] studied global gene expression profiles from 8 early stage and 8 late-stage astrocytoma patients. We calculated the overlap in the gene lists using our online Overlap tool .

## Abbreviations

**PPST: **permutation percentile separability test.

**ABA test: **a test that can detect genes with both A > B (gene is overexpressed in sample A compared to sample group B) and B < A (gene is overexpressed in sample B compared to sample group A) patterns.

**s1, s2, s3, s4: **measures of the number of samples that exhibit expression intensities beyond a specified percentile in an alternate group; used as scores in the determination of significance under the PPST and ABA tests.

## Author contributions

JLW conceived of the PPST and ABA tests and executed the analyses, SP encoded the methods in caGEDA, TG provided direction, input and scientific motivation for pursuing a test with the capabilities of the PPST and ABA tests, MJB provided, direction, input and coordination of the research. All authors read and approved the final manuscript.

## Figures and Tables

**Figure 2:**
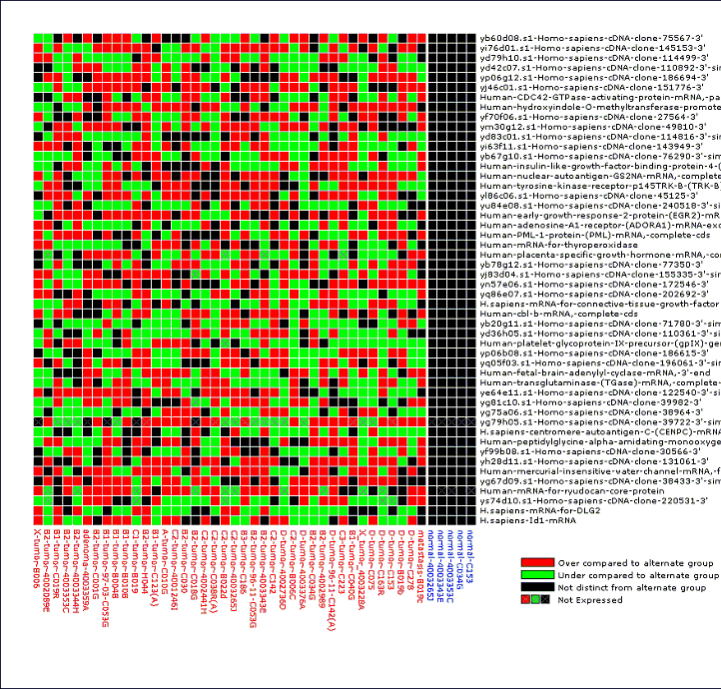
Gene Expression Pattern Grid of genes with significant ABA patterns from a comparison of epithelial-like normal colon tissue (blue samples) to colon cancers (red samples; Alon et al, 1999). We have previously determined that 5 samples in the Alon et al. data set were epithelial-like normal using unsupervised bootstrap cluster analysis and removed the remaining muscle-like normals from this analysis. These, and many other published cancer microarray data sets are 'on-tap' in our GEDA web application. The Gene Expression Pattern Grid, which is generated for any set of differentially expressed genes with the GEDA web application, summarizes the types of differential expression in a way that is related to the PPST test. Color signifies that an individual in one group exhibits an expression value that is significantly different from the expression pattern in the other group (red = overexpression; green = underexpression). Black signifies that an individual exhibits an expression value *within *the specified upper and lower percentiles in the other group. Tumor samples that fall within the upper and lower 95^th ^%tiles of the distribution of expression values from the normal samples are labeled black, showing which genes for which a sample is are not different from normal. This representation includes information on both the population-level informativeness as well as which individuals appear to exhibit uniquely differentially expressed profiles. Samples within sample group are arranged according to their relative position in a hierarchical agglomerative clustering with pairwise distance = 1-Pearson's correlation coefficient. 'Not expressed' is a hypothesis generated in these graphs when the expression intensity value of that gene for that individual falls in the lower 95^th ^%tile of the entire data set. Expression pattern grids were produced online with the Gene Expression Data Analysis web application .

**Table 1:** Number of Genes with Significant Differences between Tumor and Normal Class in Various Cancer Types under the PPST and ABA tests and the parametric t-test (for comparison)

	**α = 0.1**		**α = 0.05**		
Data Set	**PPST**	**ABA test (ABA/BAB)**	**PPST**	**ABA test (ABA/BAB)**	parametric t_α = 0.05 _**

breast^7^	572	40/5	326	28/49	313
melanoma^15^	662	60/202	312	38/133	347
colon^45^	1788	55/153	1558	46/55	1378
ovarian^16^	3344	253/63	2060	189/22	1813
lymphoma^18^	2077	286/42	1114	194/30	1370
lung^19^	614	40/3	506	35/3	389

*A = tumor sample group, B = normal sample group **pooled variance t

**Table 2:** Exemplar Genes Found to the Significant (p < 0.05) under the PPST test in the lymphoma data set^[18]^, but missed under the t-test.

**Gene**	PPST Score*	**Pattern**	**p-value under t-test**
B-cell growth factor (IL-4)	13	ABA	0.301
CCND1 Cyclin D1 (PRAD1; parathyroid adenomatosis 1)	13	AB	0.087
homeobox protein Cdx2 mRNA	12	AB	0.232
LIF Leukemia inhibitory factor (cholinergic differentiation factor)	10	AB	0.181
tumor susceptiblity protein (TSG101) mRNA	-16	BA	0.526
carcinoembryonic antigen	-18	BA	0.328
CCND2 Cyclin D2	-18	BA	0.076
VIM Vimentin	-18	BA	0.976

*PPST Score = *s*_1 _or -*s*_2 _(for AB or BA pattern) and *s*_3 _(for ABA pattern)

**Table 3:** Summary of the overlap study of the two astrocytoma progression marker data sets. A. Internal consistency of the methods under comparison. k = Khatua et al. data set^51^; vdb = van den Boom et al. data set^52 ^B. Number of significant genes that overlap between the two data sets in the significant gene list for each method. C. Comparison (% overlap) of methods in the k data set. D. Comparison (% overlap) of methods in the vdb data set

**A**	% overlap	k			
	test	t-test (p)	t-test (np)	PPST	ABA
vdb	t-test	5.307			
	t-test (np)		11.248		
	PPST			**15.24**	
	ABA				3.211
**B**	overlap	k			
vdb	test	t-test (p)	t-test (np)	PPST	ABA
	t-test	35			
	t-test (np)		118		
	PPST			**187**	
	ABA				11
**C**		Test 1			
	k	t-test (p)	t-test (np)	PPST	ABA
Test 2	t-test (p)	1	78.527	74.394	2.579
	t-test (np)		1	**83**	4.678
	PPST			1	1.28
	ABA				1
		Test 1			
**D**	v	t-test (p)	t-test (np)	PPST	ABA
Test 2	t-test (p)	1	46.382	26.649	0
	t-test (np)		1	**56.975**	5.656
	PPST			1	7.268
	ABA				1
